# Impact of laparoscopic ovarian drilling on serum anti-mullerian hormone levels in patients with anovulatory Polycystic Ovarian syndrome

**DOI:** 10.4274/tjod.97523

**Published:** 2016-12-15

**Authors:** Sobhana Paramu

**Affiliations:** 1 Lifeline Superspeciality Hospital, Clinic of Obstetrics and Gynecology, Kerala, India

**Keywords:** anti-Müllerian hormone, laparoscopic ovarian drilling, ovarian reserve, polycystic ovarian syndrome

## Abstract

**Objectives:**

Anti-mullerian hormone (AMH) is a marker of the activity of recruitable ovarian follicles. It is useful in the prediction of ovarian reserve. Women with polycystic ovarian syndrome (PCOS) have elevated circulating and intrafollicular AMH levels. Laparoscopic ovarian drilling (LOD) in patients with PCOS destroys ovarian androgen-producing tissue and reduces their peripheral conversion to estrogens. Identifying factors that determine the response of patients with PCOS to LOD will help in selecting the patients who would likely benefit from this treatment. AMH is one such marker that can predict the response to LOD. To evaluate the effect of LOD on serum AMH levels among PCOS responders and non-responders and the usefulness of AMH as a tool in predicting the response to LOD, and to whether there was loss of ovarian function after LOD.

**Materials and Methods:**

This is a prospective cohort study including 30 clomiphene-resistant women with anovulatory PCOS undergoing LOD. Statistical analysis was performed to evaluate the effect of LOD on serum levels of AMH on these women.

**Results:**

A significant fall in the levels of AMH was observed after LOD in both responders and non-responders (p<0.001). Women with AMH >8.3 ng/mL showed a significantly lower ovulation rate (33.3%). LOD was not associated with a risk of diminished ovarian reserve.

**Conclusion:**

LOD is an effective first-line treatment for women with PCOS who are clomiphene resistant. LOD has no negative effect on ovarian reserve. AMH is a useful marker in predicting the outcome of LOD.

## INTRODUCTION

Polycystic ovarian syndrome (PCOS) is one of the most common endocrine disorders of women in the reproductive age group, affecting about 4 to 12% of women worldwide^(1)^. It is characterized by a combination of hyperandrogenism (either clinical or biochemical), chronic anovulation and polycystic ovaries, and is frequently associated with insulin resistance and obesity. The underlying cause of PCOS is unknown. However, a genetic basis that is both multifactorial and polygenic is suspected, because there is well documented aggregation of the syndrome within families^(2)^.

Anti-mullerian hormone (AMH), also known as mullerian-inhibiting substance, was until recently, known mainly as a substance involved in male sexual differentiation. AMH is now considered as a marker that can estimate the quantity and activity of recruitable follicles in early stages of growth, thus being more reliable for the prediction of ovarian reserve. Women with PCOS have about 2-3 times elevated circulating and intrafollicular AMH levels. There are controversial data regarding whether the AMH excess in PCOS is related to the increment in the number of preantral follicles or due to an intrinsically increased production by granulosa cells. However, the increase may also be a consequence of other factors in PCOS such as hyperandrogenism and insulin resistance^(3,4)^. This increased AMH is associated with retardation of follicular development.

The principle behind laparoscopic ovarian drilling (LOD) in patients with PCOS is to destroy ovarian androgen-producing tissue and reduce peripheral conversion of androgens to estrogens. Specifically, a fall in serum levels of androgens and luteinizing hormone (LH) and an increase in the level of follicle-stimulating hormone (FSH) have been demonstrated after ovarian drilling^(5,6)^. The endocrine changes following surgery are thought to convert the adverse androgen dominant intra-follicular environment to one that is estrogenic, and to restore the normal hormonal environment by correcting ovarian pituitary feedback^(7)^.

Identifying factors that determine the response of women with PCOS to LOD will help in selecting the patients who are likely to benefit from this treatment, thus avoiding fruitless treatment and improving success rate. The consistency of serum levels of AMH throughout the menstrual cycle, with very little inter cycle variability, makes it an attractive marker of response to treatment. With this background, we conducted a prospective cohort study to evaluate the effect of LOD on plasma levels of AMH in PCOS.

## MATERIALS AND METHODS

This is a prospective cohort study on clomiphene citrate (CC)-resistant women with anovulatory PCOS. The study was conducted in a 300-bed super specialty obstetrics and gynecology hospital and in vitro fertilization center. The women included in the study were aged between 18 to 35 years. This study was conducted over a period of one year (April 2013 to April 2014) and included 30 women who were infertile and anovulatory with CC resistant PCOS. Each woman underwent LOD. The primary outcomes were the effect of LOD on serum AMH levels and the difference between AMH levels in responders (women who ovulated) and non-responders (no ovulation). The secondary outcomes studies were the usefulness of AMH as a tool in evaluating the outcome of LOD and to assess the ovarian reserve after LOD. PCOS was diagnosed based on the 2003 Rotterdam European Society for Human Reproduction/American Society of Reproductive Medicine criteria(8). The women were followed up for a year after undergoing LOD and evaluated regarding the response to LOD in terms of ovulation and pregnancy, and also to determine whether there was any loss of ovarian function because of LOD.

All women included in this study had normal hysteron-salpingogram and their partners had normal semen analysis according to the World Health Organization criteria^(9)^. Blood samples were collected on day 2 of the cycle before and 1 week after LOD to measure plasma concentrations of AMH, luteinizan hormon (LH), follicle stimulating hormone (FSH), testosterone (T), sex hormone-binding globulin (SHBG), and free androgen index {T/SHBG x 100}.

Additional blood samples were collected 3 and 6 months after LOD for the measurement of AMH. Plasma samples were assayed for AMH in duplicate using a commercial enzyme-linked immunosorbent assay kit (Immunotech, Beckman-Coulter UK Ltd., High Wycombe, Buckinghamshire, United Kingdom) in accordance with the manufacturer’s protocol. The sensitivity of the assay was 0.24 ng/mL. The intraassay and interassay variabilities were 5% and 8%, respectively.

Assays for LH and FSH were performed using an automated microparticle enzyme immunoassay (Abbott Axsymanalyser; Abbott Diagnostics). Assays for SHBG were performed using an automated chemiluminescent immunoassay (Immuliteanalyser; Diagnostic Products Corporation).

LOD was perfomed under general anesthesia using a monopolar electrocautery needle. Four punctures were made per ovary at a power setting of 40 W for 4-6 seconds at each point. If the patient did not ovulate after LOD, CC would be started 6-8 weeks after surgery on days 2-6 of the menstrual cycle.

Ovulation was diagnosed by serial sonographic monitoring of follicular growth and follicular collapse with elevated serum progesterone levels 7 days later. The occurrence of ovulation or clinical pregnancy during a period of 6 months were noted. Pregnancy was diagnosed through a positive quantitative β-hCG and a definite gestational sac in an ultrasound examination.

Hormonal values were expressed as mean ± standard deviation. Comparison of values before and after LOD was performed using Student’s paired t-test. Comparison of values between responders and non-responders was made using Student’s independent t-test. All p-values less than 0.05 were considered significant.

## RESULTS

**Figure 1:**
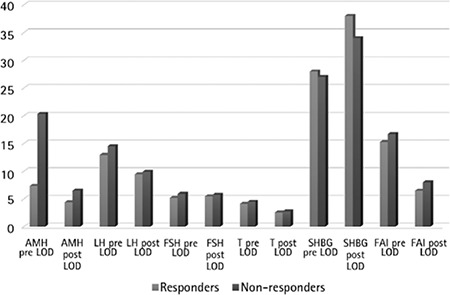
Comparison of variables among responders and non-responders before and after laparoscopic ovarian drilling *LH: Luteinizing hormone, FSH: Follicle-stimulating hormone, AMH: Anti-mullerian hormone, T: Testosterone, SHBG: Sex hormone-binding globülin, FAI: Free androgen index, LOD: Laparoscopic ovarian drilling*

The mean age of the participants was 28.3±3.5 years. The study cohort women were obese, as reflected by a high body mass index (BMI) and waist:hip ratio. Serum levels of AMH, LH, testosterone, and the LH:FSH ratio were higher in non-responders than responders before laparoscopy (Figure 1). About 80% (24/30) of women with PCOS responded to LOD as evidenced by spontaneous ovulation. The pregnancy rate was 41.66% (10/24). About 20.0% (6/30) women with PCOS were still resistant to LOD. About 58.33% (14/24) did not conceive despite the high ovulation rate (80%).

**Figure 2:**
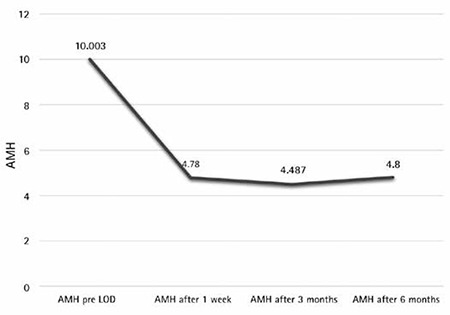
Anti-mullerian hormone values before and after laparoscopic ovarian drilling among polycystic ovarian syndrome women *AMH: Anti-mullerian hormone, LOD: Laparoscopic ovarian drilling*

Significant differences in AMH levels were observed between responders and non-responders before and after laparoscopy (p<0.001). Plasma levels of testosterone were significantly lower in responders when compared with non-responders (no ovulation). There was no significant difference in LH, LH:FSH between responders and non-responders both before and after LOD (Table 1 and 2). As a result of LOD, significant reductions in levels of AMH were observed in both responders and non-responders, but the magnitude of change was significantly higher in responders (p<0.001) when compared with non-responders (p<0.028) (Figure 2). There was no significant change in the levels of FSH in both responders and non-responders after LOD. No significant correlation was observed between plasma levels of AMH and age, BMI, LH or FSH. There was a significant positive correlation noted between plasma AMH and testosterone levels (any statistical test?). LOD led to reduced levels of AMH in patients with PCOS, but these changes were not statistically significant and only indicated the patient’s normality and had no negative impact on ovarian reserve.

A cut-off level of AMH was identified as 8.3 ng/mL, above which the chances of ovulation seemed to be significantly reduced. Women with AMH >8.3 ng/mL showed a significantly (p<0.001) lower ovulation rate (33.3%) than that of women with AMH <8.3 ng/mL (100%). Significant differences were observed in AMH levels before and after LOD, which may indicate a possible diminished ovarian reserve. Although the AMH values after LOD were found lower than those before LOD, the after values stayed higher than normal when compared with normal women without PCOS.

## DISCUSSION

The present study shows a positive correlation between plasma AMH and testosterone values in anovulatory women with PCOS. This agrees with the study conducted by Poujade O et al and many others^(10)^. This positive association is explained by the stimulatory effect of androgens on the primodial follicular growth and granulosa cell proliferation that increases AMH secretion, or the inhibitory effect of AMH on aromatase activity, leading to an increase in androgens^(10,11)^. Another cause for the increase in AMH and androgens in PCOS is secondary to hyperinsulinemia, because it enhances gonadotropin-stimulated steroid production in granulosa and theca cells. A possible explanation for the 58.33% of women with PCOS not conceiving, despite the high ovulation rate after LOD, is that the amount of ovarian tissue destroyed during LOD may not have been enough to induce favorable changes on reproductive parameters by reducing intraovarian AMH to a level consistent with resumption of ovulation. This study shows a significant decrease in serum testosterone, LH, and LH:FSH ratio, one week after LOD. These findings are in agreement with many previous studies^(12,13)^. Although the after LOD values were found lower than the before LOD values with ovarian reserve markers, the after values remained higher in non-responders when compared with the responders. Previous studies have shown that the ovarian reserve in women with PCOS was found higher than in women with normal menstruation^(13)^.

It can be deduced from earlier studies that LOD normalizes ovarian function, which is significant in follicular recruitment and maturation, and has no negative effect on ovarian reserve. It seems that the ovarian tissue damage occurs during and continues only for a short period after LOD, as evidenced by the fact that the AMH and FSH levels did not correlate with time since LOD^(13,14)^.

The reduction in AMH levels results from the bilateral diathermy technique where the androgen producing stroma is destroyed. There is a decrease in ovarian stromal blood flow and subsequently vascular endothelial growth factor and insulin-like growth factor 1, which are high in PCOS. In our study, LOD appeared not to be associated with an increased risk of diminished ovarian reserve. Most of the changes in ovarian reserve markers in the current work after LOD could be interpreted with the normalization of ovarian function in the enrolled women with PCOS rather than the reduction of ovarian reserve.

Similar to the study by Api^(15)^, we found that the ovarian reserve of patients with PCOS did not change significantly after LOD, and the reduction of AMH after LOD may be referred to as the normalization of women with PCOS after LOD. Thus, the likelihood of traumatic injury to ovaries as because of LOD is negligible. Overall, it seems that although LOD leads to a reduction in AMH levels in women with PCOS, these are not statistically significant and only indicate the patient’s normality and has no negative impact on ovarian reserve^(15)^.

## CONCLUSION

Based on the results of this study, LOD is recommended as an effective first-line treatment in women with PCOS who are anovulatory and clomiphene resistant. LOD has no negative effect on ovarian reserve, as shown by the markers of ovarian reserve such as FSH and AMH during the follow-up period. It is also recommended that women who are candidates for undergoing LOD may benefit from the measurement of serum AMH concentration to determine their likelihood of response to LOD. Women found to have high serum AMH levels (>8.3 ng/mL) can be counselled about the lower chances of responding to LOD. It is also recommended that using AMH as a reliable marker of ovarian reserve and measuring it in women with anovulatory PCOS undergoing LOD may provide a tool for predicting the outcome of LOD.

## Figures and Tables

**Table 1:**
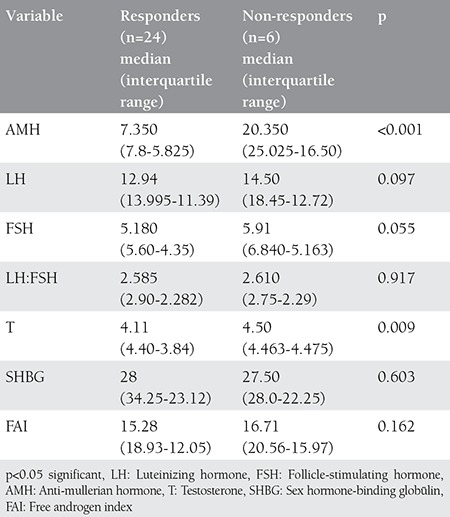
Comparison of variables between responders and non-responders before laparoscopic ovarian drillin

**Table 2:**
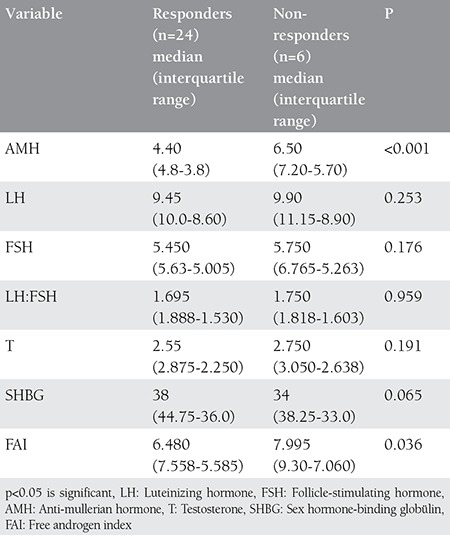
Comparison of different variables between responders and non-responders after laparoscopic ovarian drilling
